# Supplementary material for: Study protocol for a mixed methods approach to optimize colorectal cancer screening in Malaysia: Integrating stakeholders insights and knowledge-to-action framework
Source: PLoS One. 2024 Apr 9;19(4):e0299659. doi: 10.1371/journal.pone.0299659 (PMC11003698; doi:10.1371/journal.pone.0299659)
Supplement: S1 File — (DOCX) [file pone.0299659.s001.docx]

# Annex 1: Good reporting of a mixed methods study (GRAMMS) checklist

| Guideline | Section: page |
| --- | --- |
| Describe the justification for using a mixed methods approach to the research question | Mixed methods design. Page 13  Discussion. Page 24, 25 |
| Describe the design in terms of the purpose, priority, and sequence of methods | Mixed methods design. Page 13  Figure 1 |
| Describe each method in terms of sampling, data collection, and analysis | Each method (i.e., literature review, quantitative data, and qualitative data) is described based on search strategy, data sources, participant selection, data collection methods for all the methods, and data analysis for each data type.  Page 14-21 |
| Describe where integration has occurred, how it has occurred, and who has participated in it | Mixed methods design. Page 13  Data integration. Page 21 |
| Describe any limitation of one method associated with the presence of the other method | Discussion. Page 24, 25 |
| Describe any insights gained from mixing or integrating methods | Discussion. Page 24, 25 |

Source: O'Cathain A, Murphy E, Nicholl J. The quality of mixed methods studies in health services research. J Health Serv Res Policy. 2008;13(2):92-98.

# Annex 2: Data extraction form for literature review (will be built in Google sheets)

| Column heading | Input type | Options, if applicable |
| --- | --- | --- |
| Reference information | Open text | N/A |
| Publication year | Open text | N/A |
| Number of scholarly sources searched | Open text | N/A |
| Type of document | Dropdown | Original article, review, perspective/opinion/commentary, abstract, grey literature |
| Country | Open text |  |
| Intervention (screening test) | Dropdown | iFOBT, colonoscopy, two-staged approach, others |
| The rationale for the intervention | Open text | N/A |
| Study design | Dropdown | Cross-sectional, cohort, trial, review, and others |
| Who recruited/ delivered | Open text | N/A |
| Mode of delivery (how) | Open text | N/A |
| Other information  (i.e., where, when, for whom, adaptation/modification | Open text | N/A |
| Measurement for colorectal cancer screening implementation measures | Drop down across multiple columns | Acceptability, adoption, appropriateness, feasibility, fidelity, implementation cost/resources/training, intervention complexity, penetration/ linkage with other services, reach/ uptake, sustainability |
| Additional comments | Open text | N/A |

# Annex 3: List of variables for quantitative analysis

| Variable | Potential data sources |
| --- | --- |
| Individual demographics |  |
| - Age | - Routine monitoring and evaluation data of the screening program - National cancer registry - National surveys |
| - Ethnicity |  |
| - Gender |  |
| - Education |  |
| - Occupation |  |
| - Screening-related |  |
| Risk factors for colorectal cancer (e.g., symptomatic, family history, etc.) | - Routine monitoring and evaluation data of the screening program - National cancer registry - National surveys |
| - iFOBT or self-test (date bottle was given, date specimen was returned, results) |  |
| - Follow-up after positive-iFOBT (referred and attended, date of colonoscopy, colonoscopy results) |  |
| - History of iFOBT |  |
| - iFOBT done in the public or private health sector |  |
| - iFOBT results |  |
| - Colonoscopy is done following a positive |  |
| Colorectal cancer diagnosis |  |
| - Stage at diagnosis | - National Cancer Registry (colorectal cancer) |
| - Site of tumour |  |
| - Histologic findings |  |
| Social context, physical environments, and other relevant socioeconomic variables | - National survey and census data from the Ministry of Health and Department of Statistics Malaysia - Department of Town and Country Planning Other relevant departments/ ministries |

# Annex 4: Study participant selection guidance based on colorectal cancer screening participation rates and colorectal cancer burden.

|  | Examination coverage (Colonoscopy **acceptance** after positive iFOBT; %) | Hospital appointment  **adherence** rate (%) | Further assessment **participation** rate  (colonoscopy; %) | Colorectal cancer  age-standardized incidence rate (per 100,000) |
| --- | --- | --- | --- | --- |
| National average | 90 | 51 | 70 |  |
| Lower acceptance, lower adherence, lower colonoscopy participation | | | |  |
| **Sarawak** | **80** | **39** | **56** | **11.8** |
| Pahang | 86 | 50 | 62 | 12.2 |
| Melaka | 87 | 24 | 33 | 16.9 |
| Lower acceptance, higher adherence, higher colonoscopy participation | | | |  |
| **Johor** | **85** | **56** | **92** | **16.7** |
| Labuan | 59 | 67 | 70 | 19.8 |
| Kelantan | 81 | 51 | 77 | 10.4 |
| Lower acceptance, lower adherence, higher colonoscopy participation | | | |  |
| **Negeri Sembilan** | **87** | **48** | **72** | **16.1** |
| Higher acceptance, lower adherence, higher colonoscopy participation | | | |  |
| **Pulau Pinang** | **90** | **30** | **90** | **18.8** |
| Higher acceptance, lower adherence lower colonoscopy participation | | | |  |
| **JKWPKL-Putrajaya** | **94** | **44** | **66** | **13.0** |
| Higher acceptance, higher adherence, lower colonoscopy participation | | | |  |
| **Kedah** | **98** | **56** | **60** | **10.1** |
| Perlis | 91 | 61 | 57 | 11.0 |
| Perak | 92 | 67 | 68 | 12.4 |
| Higher acceptance, higher adherence, higher colonoscopy participation | | | |  |
| **Terengganu** | **91** | **54** | **90** | **13.1** |
| **Selangor** | **94** | **65** | **72** | **10.5** |
| **Sabah** | **100** | **53** | **79** | **13.2** |

Notes: The data is sourced from the Disease Control Division, the Ministry of Health (routine monitoring and evaluation data of the screening program), and the National Cancer Registry (Ministry of Health, 2019). The rates are based on preliminary analysis using national averages for participant selection. Further analysis will be conducted in the quantitative phase. The selection of potential participants (highlighted in bold) by states will be finalized in consultation with key stakeholders, and the healthcare providers’ facilities will be determined accordingly. Population participation in colorectal cancer screening suggests the presence of seven out of eight possible combinations, indicating that no consistent pattern of lower acceptance, higher adherence, and lower colonoscopy participation was observed among the states. Variations may be observed among the states with high acceptance, adherence, and participation rates. To capture these differences and gather diverse experiences, three states have been selected for inclusion in the study. Terengganu, for example, may offer valuable insights into best practices based on the experiences of healthcare providers in that region. In Sabah, where physical accessibility may pose challenges, indicators may not capture the full extent of screening implementation challenges. Meanwhile, with its high patient load, Selangor presents an opportunity for participants to share strategies and lessons learned in managing such a demanding workload.

# Annex 5: Interview guide for qualitative phased based on (a) process flow diagramming session and (b) system support mapping session (adapted from O’Leary, 2022)

1. **Process flow diagramming session**

Section 1: Stakeholder type and decision-making role

- What is your current occupation (relates to colorectal cancer screening)?

How long have you been in this role?

- What is your role in making decisions about or implementing colorectal cancer screening program at your organization/ healthcare facility?

Section 2: Introduction to process flow diagramming

- This study aims to understand the current implementation of colorectal cancer screening intervention at your facility. We will be using process flow diagrams to support the work. We will show you different versions of process flow diagrams that each describe how the same colorectal cancer screening intervention is implemented across different settings.
- We will walk through the general structure of these process flow diagrams and then use them to describe how the intervention is being implemented. This swim lane diagram shows different steps in two healthcare facilities. Please note that modifications may be made to adapt these steps to your local setting.

Section 3: Discussion on process flow diagram

- What aspects of the process flow diagram do you like? Which do you dislike? Why?
- Probes: Are the decision points relevant? Is it helpful to note the core steps? Are there other stakeholders that you feel are missing?
- What additional information did you learn from the process flow diagram? What are the similarities and differences?
- Is there anything you recommend changing about this diagram to optimize the screening implementation? If so, what?
- Based on what you have seen in the diagrams, what would implementing this intervention in your local setting take?
- Some steps are centralized/decentralized in this intervention. Which steps might need to be centralized/decentralized to best support the implementation/ your organization?
- Do you want to share anything about these diagrams and the implementation of colorectal cancer screening in your setting?

1. **System support mapping session**

Session 1: Overview of the session

- The goal is to reflect on your role and responsibilities within the colorectal cancer screening programme. Through the activity today, we ask that you consider what has worked well for you so far, what has not, and what might help improve the screening services' implementation.
- The information gathered today will be used to consider optimizing the current implementation and delivery of services.
- To help us think about your roles in implementing the screening intervention, we will use a system support mapping tool. We are going to do this using paper and sticky notes.
- System support mapping is designed to help with systems thinking and consider complex problems. We will use this systems approach to help us understand the implementation and sustainment of colorectal cancer services.
- By doing so, we can consider how you break down your work into specific responsibilities given each of your roles.
- For each responsibility, what do you need in order to be successful? By looking across your maps, we can better describe the puzzle pieces, clearly document implementation needs, and prioritize our efforts to strengthen the screening program.

Session 2: Walk through an example of system support mapping.

- We will present an example of a system support map to completed. The way this is structured is that there are multiple rings or concentric circles, with sticky notes in each ring.
- You will fill out these sticky notes based on your work experiences implementing the colorectal cancer intervention.
- We are gradually going through this process of creating a map, but we wanted to show you a snapshot of what a completed map will look like first.
- We will walk through each of the rings to indicate how you should create your map and provide a more simplified example.

Roles in the first/center ring

- You should use a blue sticky note to describe your identity or primary role in implementing the colorectal cancer program. This section should be a brief description.

Responsibilities for implementing the screening program.

- The second ring is for noting your responsibilities involved in implementing the program. You can think of responsibilities as the discrete activities you must perform to carry out your role. Your responsibilities can be activities related to any aspect of the intervention – from identifying a patient eligible for screening to navigating a patient through a follow-up colonoscopy.
- Report each discrete activity or responsibility on a separate green sticky note. Then, select the four most complex or involved activities and include them in this ring. The others can be listed in the corner of the paper to provide the full scope of your work activities.

Needs in the third ring.

- You should consider – what do I need in order to perform each of these responsibilities? For instance, you can think about skills, buy-in, materials, types of interactions, electronic resources, training, logistical needs, or anything else required to perform each of the responsibilities you previously identified.
- Use yellow sticky notes, reporting one need per sticky note. Please identify your three most critical needs for each responsibility.

Resources you have used in the fourth ring.

- Here are the resources you have used to address your identified needs. Examples of types of resources you may have consulted or used include training, technical assistance, documentation, and websites. You should identify any resources you have consulted to perform your responsibilities.
- Use pink notes for resources. Each note can contact as many resources as you need. For each resource listed, please note whether you found that resource to be helpful or unhelpful (yes or no) in addressing your need.

Suggestions in the fifth ring

- You will make suggestions for improvement using the orange sticky notes. You can think about your suggestions for particular resources that would be useful, such as ways to streamline your work processes or ways to improve the implementation of the intervention more broadly.
- There are no limits – you can be ambitious about what might strengthen your ability to carry out your responsibilities or the implementation team’s ability to implement the screening program efficiently. Each of us gets four suggestions overall, which can relate to any of your responsibilities. For example, you might have one suggestion per responsibility or multiple suggestions for two or three responsibilities.

Contextual factors affecting them or the intervention in the final ring/ space.

- The outer ring or empty spaces report contextual factors shaping your approach to a particular responsibility.
- There might be differences in implementing the intervention in specific settings, e.g., geographic locations and patient populations. Report each factor per blue sticky notes.

Session 3: Participants create their maps.

- To create your maps, we encourage you to be as detailed as possible in your map so that it can stand alone and be understood.
- You will have about [40 minutes] to create your map.
- As you make your map, please let us know if you have any questions or just want to talk through specific sticky notes or rings.
- During the remaining time, we will take a few minutes for each person to share their map, particularly describing the responsibilities listed and how you arrived at the suggestions you made. This section will be a chance to do informal storytelling about what you included and why.

Session 4: Participants reflect on their maps.

- Since our session has some diverse perspectives, it can be helpful to learn from each other. We are most interested in having you talk through the responsibilities you listed in your map and then explain why you selected the four suggestions you chose. You can think of this as telling the story behind how you settled on each suggestion. Feel free to work outward across the rings or from the outer ring to the inner ring.
- As the others share their maps, please feel free to chime in with any questions or comments. For example, do you have any takeaways from seeing and hearing about their map? Is there anything you think they missed in their map (e.g., another activity they might perform) or anything you want further clarification? [followed by debriefing session]
- With our last few minutes, do you have any comments or takeaways about this process more broadly?

That concludes our discussion today. Thank you for your participation and reflection during our session. The maps you created will be reviewed in more detail and used to inform the optimization strategies to improve the colorectal cancer screening program.
