# Supplementary material for: Study protocol for a mixed methods approach to optimize colorectal cancer screening in Malaysia: Integrating stakeholders insights and knowledge-to-action framework
Source: PLoS One. 2024 Apr 9;19(4):e0299659. doi: 10.1371/journal.pone.0299659 (PMC11003698; doi:10.1371/journal.pone.0299659)
Supplement: Attachment — Submitted filename: PONE-D-23-33266.pdf [file pone.0299659.s002.pdf]

PLOS ONE

Study protocol for a mixed methods approach to optimize colorectal cancer screening in Malaysia: Integrating stakeholders insights and knowledge-to-action framework.

--Manuscript Draft--

**Manuscript Number:**	PONE-D-23-33266
**Article Type:**	Study Protocol
**Full Title:**	Study protocol for a mixed methods approach to optimize colorectal cancer screening in Malaysia: Integrating stakeholders insights and knowledge-to-action framework.
**Short Title:**	Optimizing colorectal cancer screening in Malaysia: A mixed-methods protocol
**Corresponding Author:**	Diane Woei-Quan Chong<br>Institute for Health Systems Research: Institut Penyelidikan Sistem Kesihatan<br>Shah Alam, Selangor MALAYSIA
**Keywords:**	Colorectal cancer; screening; mixed methods; health systems; systems approach; knowledge-to-action
**Abstract:**	Introduction<br><br>Colorectal cancer is a growing global health concern and the number of reported cases has increased over the years. Early detection through screening is critical to improve outcomes for patients with colorectal cancer. In Malaysia, there is an urgent need to optimize the colorectal cancer screening program as uptake is limited by multiple challenges. This study aims to systematically identify and address gaps in screening service delivery to optimize the Malaysian colorectal cancer screening program.<br><br>Methods<br><br>This study uses a mixed methods design. It focuses primarily on qualitative data to understand processes and strategies and to identify specific areas that can be improved through stakeholder engagement in the screening program. Quantitative data play a dual role in supporting the selection of participants for the qualitative study based on program monitoring data and assessing inequalities in screening and program implementation in healthcare facilities in Malaysia. Meanwhile, literature review identifies existing strategies to improve colorectal cancer screening. Additionally, the knowledge-to-action framework is integrated to ensure that the research findings lead to practical improvements to the colorectal cancer screening program.<br><br>Discussion<br><br>Through this complex mix of qualitative and quantitative methods, this study will explore the complex interplay of population- and systems-level factors that influence screening rates. It involves identifying barriers to effective colorectal cancer screening in Malaysia, comparing current strategies with international best practices, and providing evidence-based recommendations to improve the local screening program.
**Order of Authors:**	Diane Woei-Quan Chong
	Vivek Jason Jayaraj
	Fathullah Iqbal Ab Rahim
	Sharifah Saffinas Syed Soffian
	Muhammad Fikri Azmi
	Mohd Yusaini Mohd Yusri
	Ahmad Shanwani Mohamed Sidek
	Norfarizan Azmi
	Rosaida Md Said

Powered by Editorial Manager® and ProduXion Manager® from Aries Systems Corporation

	Muhammad Firdaus Md Salleh
	Norasiah Abu Bakar
	Hamiza Shahar
	Rima Marhayu Abdul Rashid
	Shazimah Abdul Samad
	Zanita Ahmad
	Mohd Safiee Ismail
	Adilah A. Bakar
	Nor Mashitah Hj Jobli
	Sondi Sararaks
**Additional Information:**	
**Question**	**Response**
**Financial Disclosure**<br><br>Enter a financial disclosure statement that describes the sources of funding for the work included in this submission. Review the submission guidelines for detailed requirements. View published research articles from *PLOS ONE* for specific examples.<br><br>This statement is required for submission and **will appear in the published article** if the submission is accepted. Please make sure it is accurate.<br><br>**Funded studies**<br>Enter a statement with the following details:<br>• Initials of the authors who received each award<br>• Grant numbers awarded to each author<br>• The full name of each funder<br>• URL of each funder website<br>• Did the sponsors or funders play any role in the study design, data collection and analysis, decision to publish, or preparation of the manuscript?<br><br>Did you receive funding for this work?	Yes
Please add funding details.<br>as follow-up to "**Financial Disclosure**<br><br>Enter a financial disclosure statement that describes the sources of funding for the	Funding for this research has been applied for through the Ministry of Health Malaysia Research Grant (MRG), with approval pending. The prospective funding body will have no involvement in the study design, data collection and analysis, publication decision, or manuscript preparation.

work included in this submission. Review the submission guidelines for detailed requirements. View published research articles from *PLOS ONE* for specific examples.<br><br>This statement is required for submission and **will appear in the published article** if the submission is accepted. Please make sure it is accurate.<br><br>**Funded studies**<br>Enter a statement with the following details:<br>• Initials of the authors who received each award<br>• Grant numbers awarded to each author<br>• The full name of each funder<br>• URL of each funder website<br>• Did the sponsors or funders play any role in the study design, data collection and analysis, decision to publish, or preparation of the manuscript?<br><br>Did you receive funding for this work?"	
Please select the country of your main research funder (please select carefully as in some cases this is used in fee calculation).<br>as follow-up to "**Financial Disclosure**<br><br>Enter a financial disclosure statement that describes the sources of funding for the work included in this submission. Review the submission guidelines for detailed requirements. View published research articles from *PLOS ONE* for specific examples.<br><br>This statement is required for submission and **will appear in the published article** if the submission is accepted. Please make sure it is accurate.	MALAYSIA - MY

**Funded studies**<br>Enter a statement with the following details:<br>• Initials of the authors who received each award<br>• Grant numbers awarded to each author<br>• The full name of each funder<br>• URL of each funder website<br>• Did the sponsors or funders play any role in the study design, data collection and analysis, decision to publish, or preparation of the manuscript?<br><br>Did you receive funding for this work?"	
**Competing Interests**<br><br>Use the instructions below to enter a competing interest statement for this submission. On behalf of all authors, disclose any competing interests that could be perceived to bias this work—acknowledging all financial support and any other relevant financial or non-financial competing interests.<br><br>This statement is required for submission and **will appear in the published article** if the submission is accepted. Please make sure it is accurate and that any funding sources listed in your Funding Information later in the submission form are also declared in your Financial Disclosure statement.<br><br>View published research articles from *PLOS ONE* for specific examples.	The authors have declared that no competing interests exist.

**NO authors have competing interests**<br><br>Enter: *The authors have declared that no competing interests exist.*<br><br>**Authors with competing interests**<br><br>Enter competing interest details beginning with this statement:<br><br>*I have read the journal's policy and the authors of this manuscript have the following competing interests: [insert competing interests here]*<br><br>* typeset	
**Ethics Statement**<br><br>Enter an ethics statement for this submission. This statement is required if the study involved:<br><br>• Human participants<br>• Human specimens or tissue<br>• Vertebrate animals or cephalopods<br>• Vertebrate embryos or tissues<br>• Field research<br><br>Write "N/A" if the submission does not require an ethics statement.<br><br>General guidance is provided below. Consult the submission guidelines for detailed instructions. **Make sure that all information entered here is included in the Methods section of the manuscript.**	The study will comply with ethical principles outlined in the Declaration of Helsinki and Malaysian Good Clinical Practice Guideline. Ethical approval was obtained from the Medical Research and Ethics Committee, Ministry of Health (23-02197-KTT). The study is registered at the National Medical Research Register (NMRR ID-23-02197-KTT).

Powered by Editorial Manager® and ProduXion Manager® from Aries Systems Corporation

**Format for specific study types**<br><br>**Human Subject Research (involving human participants and/or tissue)**<br>• Give the name of the institutional review board or ethics committee that approved the study<br>• Include the approval number and/or a statement indicating approval of this research<br>• Indicate the form of consent obtained (written/oral) or the reason that consent was not obtained (e.g. the data were analyzed anonymously)<br><br>**Animal Research (involving vertebrate animals, embryos or tissues)**<br>• Provide the name of the Institutional Animal Care and Use Committee (IACUC) or other relevant ethics board that reviewed the study protocol, and indicate whether they approved this research or granted a formal waiver of ethical approval<br>• Include an approval number if one was obtained<br>• If the study involved *non-human primates*, add *additional details* about animal welfare and steps taken to ameliorate suffering<br>• If anesthesia, euthanasia, or any kind of animal sacrifice is part of the study, include briefly which substances and/or methods were applied<br><br>**Field Research**<br><br>Include the following details if this study involves the collection of plant, animal, or other materials from a natural setting:<br>• Field permit number<br>• Name of the institution or relevant body that granted permission	
**Data Availability**<br><br>Authors are required to make the data underlying their research findings fully available, without restriction.<br><br>If the manuscript reports pilot or preliminary results, the data underlying those results must be made available at the time of publication. A Data Availability	No - Results are reported and there are restrictions to sharing the data

Statement describing where the data can be found is required at submission. Your answers to this question constitute the Data Availability Statement and will be published in the article, if accepted. When legal or ethical requirements prohibit public data sharing, authors must indicate how researchers may obtain access to the data. See the PLOS Data Policy and FAQ for detailed information.<br><br>**Important:** Stating 'data available on request from the author' is not sufficient. If your data are only available upon request, select 'No' for the first question and explain your situation in the text box.<br><br>For protocols without pilot or preliminary data, authors are strongly encouraged to state how they plan to share research data from their study when it is completed or published.<br><br>If your study includes pilot data, will they be made fully available without restriction?	
**Describe where the data may be found in full sentences. If you are copying our sample text, replace any instances of XXX with the appropriate details.**<br><br>• If **no pilot data are reported** in the manuscript, please enter the following: *No datasets were generated or analysed during the current study. All relevant data from this study will be made available upon study completion.*<br>• If the manuscript reports pilot data **held in a public repository**, include URLs, accession numbers or DOIs. If this information will only be available after acceptance, indicate this by ticking the box below. For example: *All XXX files are available from the XXX database (accession number(s) XXX, XXX.).*<br>• If the manuscript reports pilot data contained **within the manuscript and/or**	Data used in this study are sourced from the Ministry of Health Malaysia and are not publicly available due to confidentiality restrictions. However, data can be accessed upon reasonable request and subject to approval from the relevant authority within the Ministry of Health Malaysia.

**Supporting Information files**, enter the following:<br>*All relevant data are within the manuscript and its Supporting Information files.*<br>• If neither of these applies but you are able to provide **details of access elsewhere**, with or without limitations, please do so. For example:<br><br>*Data cannot be shared publicly because of [XXX]. Data are available from the XXX Institutional Data Access / Ethics Committee (contact via XXX) for researchers who meet the criteria for access to confidential data.*<br><br>*The data underlying the results presented in the study are available from (include the name of the third party and contact information or URL).*<br>• This text is appropriate if the data are owned by a third party and authors do not have permission to share the data.<br><br>For study protocols with data management or sharing plans, authors are encouraged to briefly describe in their Data Availability Statement how data they generate will be made accessible when the study is completed. For example:<br>• Deidentified research data will be made publicly available when the study is completed and published.<br><br>* typeset	
Additional data availability information:	

Powered by Editorial Manager® and ProduXion Manager® from Aries Systems Corporation

Manuscript

Click here to access/download;Manuscript;crcProtocol_v6.1_241023_submit

Study protocol for a mixed methods approach to optimize colorectal cancer screening in Malaysia: Integrating stakeholders insights and knowledge-to-action framework.

Short title

Optimizing colorectal cancer screening in Malaysia: A mixed-methods protocol

Diane Woei-Quan Chong[1], Vivek Jason Jayaraj[2], Fathullah Iqbal Ab Rahim[3], Sharifah Saffinas Syed Soffian[4], Muhammad Fikri Azmi[5], Mohd Yusaini Mohd Yusri[6], Ahmad Shanwani Mohamed Sidek[7], Norfarizan Azmi[8], Rosaida Md Said[9], Muhammad Firdaus Md Salleh[10], Norasiah Abu Bakar[11], Hamiza Shahar[12], Rima Marhayu Abdul Rashid[13], Shazimah Abdul Samad[13], Zanita Ahmad[13], Mohd Safiee Ismail[13], Adilah A. Bakar[14], Nor Mashitah Hj Jobli[14], Sondi Sararaks[15]

[1]Centre for Health Services Research, Institute for Health Systems Research, National Institutes of Health, Ministry of Health Malaysia, 40170 Shah Alam, Malaysia.

[2]Sector for Biostatistics and Data Repository, NIH Manager's Office, National Institutes of Health, Ministry of Health Malaysia, 40170 Shah Alam, Malaysia.

[3]Centre for Health Equity Research, Institute for Health Systems Research, National Institutes of Health, Ministry of Health Malaysia, 40170 Shah Alam, Malaysia.

[4]Kota Setar Health District Office, Kedah State Health Department, Ministry of Health Malaysia, 05100 Alor Setar, Malaysia.

[5]Disease Control Division, Ministry of Health Malaysia, 62590 Putrajaya, Malaysia.

[6]Bandar Sri Jempol Health Clinic, Ministry of Health Malaysia, 72120 Bandar Seri Jempol, Negeri Sembilan, Malaysia.

[7]Department of General Surgery, Hospital Raja Perempuan Zainab II, Ministry of Health Malaysia, 15586 Kota Bahru, Kelantan, Malaysia.

[8]Department of General Surgery, Hospital Tuanku Ja'afar, Ministry of Health Malaysia, 70300 Seremban, Negeri Sembilan, Malaysia.

[9]Department of Medicine, Hospital Serdang, Ministry of Health Malaysia, 43000 Kajang, Selangor, Malaysia.

[10]Department of Medicine, Hospital Sultanah Aminah, Ministry of Health Malaysia, 80100 Johor Bahru, Johor, Malaysia.

[11]Department of Medicine, Hospital Raja Perempuan Zainab II, Ministry of Health Malaysia, 15586 Kota Bahru, Kelantan, Malaysia.

[12]Department of Medicine, Hospital Tengku Ampuan Rahimah, Ministry of Health Malaysia, 41200 Klang, Selangor, Malaysia.

[13]Family Health Development Division, Ministry of Health Malaysia, 62590 Putrajaya, Malaysia

[14]Medical Development Division, Ministry of Health Malaysia, 62590 Putrajaya, Malaysia

[15]Director's Office, Institute for Health Systems Research, National Institutes of Health, Ministry of Health Malaysia, 40170 Shah Alam, Malaysia.

Corresponding author

Diane Woei-Quan Chong

Centre for Health Services Research,

Institute for Health Systems Research,

Ministry of Health Malaysia,

40170 Shah Alam, Malaysia

Tel: +60.3.33628005

Email: chong.dwq@moh.gov.my

Keywords: colorectal cancer, screening, mixed methods, health systems, systems approach, knowledge-to-action

Abstract

Introduction: Colorectal cancer is a growing global health concern and the number of reported cases has increased over the years. Early detection through screening is critical to improve outcomes for patients with colorectal cancer. In Malaysia, there is an urgent need to optimize the colorectal cancer screening program as uptake is limited by multiple challenges. This study aims to systematically identify and address gaps in screening service delivery to optimize the Malaysian colorectal cancer screening program.

Methods: This study uses a mixed methods design. It focuses primarily on qualitative data to understand processes and strategies and to identify specific areas that can be improved through stakeholder engagement in the screening program. Quantitative data play a dual role in supporting the selection of participants for the qualitative study based on program monitoring data and assessing inequalities in screening and program implementation in healthcare facilities in Malaysia. Meanwhile, literature review identifies existing strategies to improve colorectal cancer screening. Additionally, the knowledge-to-action framework is integrated to ensure that the research findings lead to practical improvements to the colorectal cancer screening program.

Discussion: Through this complex mix of qualitative and quantitative methods, this study will explore the complex interplay of population- and systems-level factors that influence screening rates. It involves identifying barriers to effective colorectal cancer screening in Malaysia, comparing current strategies with international best practices, and providing evidence-based recommendations to improve the local screening program.

Introduction

Global overview and Malaysia’s colorectal cancer landscape

Colorectal cancer is a significant global health concern with high morbidity and mortality rates. It accounts for 10% of global cancer incidence and 9.4% of cancer-related deaths. The number of people affected by colorectal cancer is influenced by human development,

References 79-80

population growth, and aging. This number is estimated to rise to 3.2 million by 2040 [1]. Early detection through effective screening programs is critical for identifying precancerous or early-stage cancer for timely intervention and treatment. The effectiveness of such screening programs in reducing morbidity and mortality associated with colorectal cancer is well-documented [2,3]. In countries with high screening uptake, such as the Netherlands, which has a 64.8% coverage, there has been a significant decrease in colorectal cancer-related morbidity and mortality, underscoring the imperative of widespread screening implementation [4–6].

References 88-91

Colorectal cancer is the second most common cancer in Malay[illegible] 13.5% of all new cancer cases diagnosed between 2012 and 2016. The incidence of colorectal cancer increases with age and is slightly higher in men (14.8/100,000) than women (11.1/100,000). Although colorectal cancer is highly preventable and treatable through early detection, approximately 70% [illegible]tients in Malaysia were diagnosed at stage III or IV [7]. The Mini[illegible]d its colorectal cancer screening program in 2014 to address th[illegible]ses an immunological fecal occult blood test (iFOBT), followed [illegible]rgets asymptomatic individuals between 50 and 75. By 2020, 598 Ministry of Health clinics are providing this service [8]. However, resource cons[illegible]e resulted in phased implementation, with less than 1[illegible]age group undergoing iFOBT screening annually (here[illegible]erage), and only 60% of those referred for colonoscopy af[illegible]proceed with the procedure in the public health sector [refer to as further assessment participation rates; 9].

Is this date correct - it is in the past.

Should be ‘referred to as…’

D[illegible]e and demonstrated effectiveness of colorectal canc[illegible]val through early diagnosis, the current scree[illegible], with participation rates falling short of intern[illegible]ited States Multi-Society Task Force on Colorectal Cancer and the European Colorectal Cancer Screening Guidelines recommend screening

This reference is from 2012. Is there a more recent recommendation from this body?

rates of at least 60% and 45-65%, respectively, for screening programs based on fecal immunochemical-based testing [10,11]. Among countries in the Asia Pacific region with high prevalence and mortality rates, Japan had an examination coverage of 11%, and Korea had a rate of 29.2%. Further assessment participation rates for these countries were 68.5% for Japan and 33% for Korea [6]. The significant discrepancy between current colorectal cancer screening rates in Malaysia and international benchmarks highlights the urgent need to improve the screening program significantly.

References
for 116-120

Sy[illegible]es: Screening implementation and socioeconomic barriers

[illegible]e screening can be multifaceted and stem from systemic deficiencies a[illegible]nequalities. Colorectal cancer screening programs rely on individual [illegible]rticipa[illegible]nd a well-functioning healthcare system that can effectively deliver screening services. Efficient and streamlined wor[illegible]tial for a successful screening program. However, variations may oc[illegible]s in the implementation of the screening program, which includes m[illegible]and phases, such as promotion of the iFOBT, screening test administrati[illegible]dent referral for colonoscopy [12–14]. The lack of integration within the healthcare systems adds complexity to the screening process and complicates coordination and continuity of care. Communication gaps, unclear responsibilities, and inadequate follow-up mechanisms hinder the seamless flow of the screening process and result in missed opportunities for early detection and timely intervention [6,15–18]. Additionally, limited access to colonoscopy services, long waiting times, and lack of coordination between primary care and specialist physicians can lead to delays in timely completion of the diagnostic phase [17,18].

Grammar
check
needed

It is also crucial to recognize the significant impact of socioeconomic inequalities on screening rates because early screening can influence disease incidence, prevalence, and outcomes [19–22]. Studies have identified several socioeconomic barriers, such as lower educational attainment, income inequality, and limited access to healthcare facilities, contributing to lower screening rates in disadvantaged populations [23,24]. It is important to

recognize that these socioeconomic factors may intersect and influence each other (e.g., individuals with lower education levels may also face financial constraints and limited access to healthcare facilities), exacerbating challenges for disadvantaged populations. By implementing comprehensive strategies that consider multiple socioeconomic factors and their interactions, health systems can improve screening rates among underprivileged populations, reduce disparities, and ensure e[illegible]ng services. This, in turn, can lead to early detection of colore[illegible]lower colorectal cancer morbidity and mortality in socioec[illegible]ions.

> Isn't this text about addressing the cultural nuances, not going beyond them?

Focus on Malaysia: Beyond the cultural fabric.

While global studies provide valuable insights, addressing colorectal cancer in Malaysia requires a holistic approach tailored to its unique challenges [25]. Malaysia's cultural diversity results in varied perceptions and beliefs regarding colorectal cancer and its screening [12–14]. Additionally, potential inconsistencies in the distribution and delivery of colorectal cancer screening in clinics and hospitals may influence the program's effectiveness. To address these nuances, we will conduct a comprehensive analysis of the Malaysian colorectal cancer screening program to pinpoint specific areas where improvements are needed. This will be achieved through the following specific objectives:

Specific objective 1: To identify strategies to increase colorectal cancer screening uptake and reduce socioe[illegible]oscopy screening.

Specific ob[illegible]l cancer screening needs and he[illegible]ctors to optimize colore[illegible]

Specific[illegible]l cancer screening program a[illegible]component colorectal screeni[illegible]n.

> What is needed here is an indication of whether there is any existing literature providing an analysis of the Malaysian screening program so that readers can understand whether this study fills a gap in the literature or builds on existing literature in a more comprehensive way.

Methods

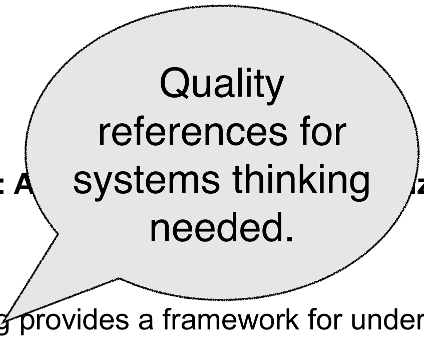

Applying systems thinking: A [illegible]ze colorectal cancer screening.

In this study, systems thinking provides a framework for understanding and improving colorectal cancer screening program. The focus shifts from isolated components of the screening process to the broader system [26]. It is recognized that the colorectal cancer screening program is not isolated but is embedded in larger healthcare systems. This perspective recognizes that the success of the screening program depends on the interactions and relationships between various levels of healthcare and stakeholders involved, including individuals, healthcare organizations, communities, and policymakers. Each level influences and is influenced by others, creating a dynamic and interconnected healthcare system [27]. By recognizing the interconnectedness of different levels of healthcare systems and stakeholders, considering the dynamic nature of the system, and identifying leverage points for interventions, systems thinking enables a comprehensive assessment of the screening program as a basis for developing effective strategies that address system-level challenges and improve colorectal cancer screening outcomes [28,29].

Mixed methods design

A mixed-methods design is used in this study. We describe the components of our study methodology using the Good Reporting of a Mixed Methods Study (GRAMMS) checklist [30, see Annex 1 in S1 File]. The primary data will be qualitative, delving deep into understanding the intricate processes, strategies for improvement, and stakeholder involvement underpinning the screening program. This data will provide valuable insights into the specific areas in need of improvement (Objectives 3).

Simultaneously, the study will utilize quantitative data in a secondary role, which serves two critical purposes. First, the quantitative data obtained from routine monitoring and evaluation of the screening program will be used sequentially to select participants for the qualitative component. This data will ensure that the qualitative phase is underpinned by

empirical evidence from the program's operational data. Next, the study takes on a concurrent approach in which the quantitative data is used to examine inequality in access to screening services and to identify differences in program implementation across healthcare settings, as outlined in Objective 2.

Meanwhile, literature review serves as the fundamental step in identifying strategies from the existing literature to improve colorectal cancer screening (Objective 1). Through this complex mix of qualitative and quantitative methods, we aim to explore the complex interplay of population- and system-level factors that influence [illegible] strategies to align the program with international best pra[illegible] study design. While the study design emphasizes the pri[illegible] secondary importance of quantitative data, and the fundam[illegible] clarity and consistency with the research objectives, the methods are described in the order of literature review, quantitative analysis, followed by qualitative analysis.

Delete the comma - it's a complicated sentence and the comma here breaks up the flow of the meaning

Fig 1. Overall study design for colorectal cancer screening research.

Legend: The central overlapping circles denote the primary qualitative and embedded quantitative data sources. Above this is a rectangle representing the fundamental literature review. The methodological flow of this study is delineated as (1) literature review, (2) quantitative analysis, and (3) qualitative analysis. A larger rectangular surrounding these methods represents the knowledge-to-action cycle. Each segment within this framework represents specific stages of the action process. Key findings from the methods that feed into each phase are highlighted under each phase. Although monitoring and refinement are not detailed in this protocol, they are presented to illustrate the entire cycle from knowledge to action.

Literature review

Purpose and rationale

The review seeks to systematically map and summarize the existing knowledge on colorectal cancer screening strategies, socioeconomic influences, reported opportunities and challenges, and stakeholder involvement. The review will contribute towards prioritizing strategies or interventions that have demonstrated positive outcomes in similar socio-cultural and healthcare settings. The list of strategies will be discussed with participants during a workshop session. The strategies will undergo further deliberation and review by key stakeholders after the workshop (see Qualitative data).

Search strategy

The search will include databases such as PubMed, EMBASE, CINAHL, and Google Scholar. The search will be conducted from September to December 2023, and subsequent updates will be performed to identify any new relevant literature. In addition to the electronic database, we will search the reference lists of identified studies to identify additional relevant sources. Google Web will also be searched to identify any pertinent unpublished reports or grey literature that may provide valuable insights for the review.

The review will include grey literature on screening programs and strategies to improve colorectal cancer screening uptake in different countries, including reports on policies, programs, development plans, and stakeholders involved. Data sources will include reports on interventions, policy documents, strategic plans, and operational documents. This approach ensures a comprehensive understanding of colorectal cancer screening practices, strategies, and stakeholder involvement.

The search terms will include key concepts such as “colorectal cancer”, “screening”, “socioeconomic factors”, “inequalities”, “access”, “utilization”, “Malaysia”, “low- and middle-income countries”, “high-income countries”, “stakeholders”, and “strategies”. The search string will be modified and adapted based on the specific databases that will be searched to ensure comprehensive coverage of the relevant literature.

Study selection

Documents will be included if they meet these criteria: (a) colorectal cancer screening intervention targeting the general adult population at risk of colorectal cancer (population), (b) interventions using iFOBT and colonoscopy (strategies), (c) set in or focused on Malaysia, low- and middle-income countries or high-income countries (context), and (d) documents and literature types such as empirical studies, commentaries, editorials, and policy reviews, grey literature, and review documents. Studies designed to improve cancer screening in cancer patients and health professionals are excluded, as are intervention protocols if do not include implementation information.

Data extraction and analysis

The study employs a [illegible] approach with an initial assessme[illegible] abstracts f[illegible] at least two independe[illegible] a pilot [illegible]mplemented to refine [illegible] Durin[illegible] established frame[illegible] Temp[illegible]hecklist with tools such as Microsoft Excel an[illegible] in Annex 2 in S1 File).

I am not qualified to comment on quantitative methods and tools, however I have reviewed this section of the text for meaning and logic in contxt to the overall study design.

Consider using Rayyan

Quantitative data collection and analysis

Purpose and rationale

We aim to understand how colorectal cancer screening rates change across regions and time and what socioeconomic factors might influence these changes. It involves (a) examining the temporal trends in colorectal cancer screening examination coverage and further assessment participation, (b) determining the spatiotemporal associations of colorectal cancer screening with the socioeconomic gradient, healthcare needs, and ecobiosocial factors in Malaysia, and (c) forecasting the screening needs of populations based on predicted cancer screening rates and the effect modification of the socioeconomic gradient.

The cornerstone of this approach lies in the fact that inequalities exist within the incidence and screening of cancer, with gradient-like differentials across space and time. These are fundamentally important in predicting healthcare needs within populations. If these estimations or predictions can be made, scenarios can be simulated to inform the allocation of resources within national-level colorectal cancer screening policy.

Study design and study scope

A spatiotemporal study will be implemented to examine the associations between the incidence of colorectal cancer screening and area-based indicators of socioeconomic position, which proxy as the upstream determinants of cancer risk factors. This design is chosen because it can capture the dynamic nature of colorectal cancer screening rates over time and allows for various factors' impact across different regions. The study population will include all populations in Malaysia screened for and diagnosed with colorectal cancer in any healthcare facility in Malaysia. The study outcome is colorectal cancer screening rates.

Data sources and variables

This study will utilize registry-derived measures of colorectal cancer screening rates in public primary care facilities, census and survey-derived area-based socioeconomic measures, geographic information systems (GIS) data-derived environmental characteristics, and area-based healthcare access characteristics in addressing the study objective. These secondary data will be aggregated from several sources, including routine monitoring and evaluation data of the screening program, national cancer registry, national surveys, and census data from the Department of Statistics Malaysia.

Individual and community/ population-level characteristics (indicating demand). Screening data and colorectal cancer incidence will be sourced from the screening program and self-reported entries in national surveys and the national cancer registry. Socioeconomic indicators will be taken from national surveys and the Department of Statistics Malaysia.

Information on physical environments, such as open spaces and land use, will be sourced from the Department of Town and Country Planning.

Screening services at facility or regional levels (indicating supply). Data will include a list of healthcare providers and facilities, especially primary care clinics and their respective referral hospitals. The expansion of colorectal cancer screening services over time will also be examined. A summary of variables that may be included and data sources is provided in Annex 3 in S1 File.

Data analysis

Descriptive analysis will be conducted to examine trends and changes in screening rates. Age-standardized rates will be determined and mapped geographically, with visual representations illustrating regional differences. The average annual percentage of change will be estimated to analyze the trends in screening rates over time. It will be analyzed using the piecewise log-linear function of the trend under assumptions of non-linearity within the rate of change [31,32].

Spatial autocorrelation tests, including Moran's I coefficient and Geary's ratio, will measure the clustering effect in the screening rates over the study duration. Associations between colorectal cancer screening rates and identified factors, such as healthcare resources and ecobiosocial variables, will be analyzed using spatial regression. Specific models within this framework will be selected depending on data characteristics, potentially assuming a Poisson distribution for count or rate data. Additionally, Bayesian spatial regression will be applied using the R-INLA method, with model priors informed by prior knowledge and data nature, detailing hyperparameters and spatial structures, such as the Besag-York-Mollié model. The model specifics, encompassing interaction or higher-order terms, will be refined through preliminary analyses and theoretical underpinnings. Diagnostic checks for spatial and Bayesian spatial regressions will be performed to ascertain model

integrity, including residual analysis, spatial autocorrelation checks, and convergence diagnostics like the R-hat statistic for the Bayesian approach.

Variations in screening rates by region will be determined using the most accurate predictive model, and scenario analysis will be employed to observe the potential shifts in screening rates upon altering healthcare resource availability. Sensitivity analyses will be carried out by including and excluding variables to understand the model in greater detail. All data analyses will be conducted using R (version 4.2.2).

Qualitative data collection and analysis

Purpose and rationale

This study will employ an exploratory qualitative approach to gather stakeholder-specific roles, responsibilities, and critical factors influencing the implementation of colorectal cancer screening intervention in Malaysia. It will involve the development of process flow diagrams and system-support mapping, which will visually depict the process steps and explore how these steps are implemented across different regions, thus capturing procedural aspects and factors influencing the implementation of the colorectal cancer screening program.

Data collection methods

A workshop-style approach will be implemented to explore the participants' perspectives, experiences, and challenges faced during the implementation of the screening intervention. We will examine and map out different steps and processes, identify variations and gaps, and propose improvements in implementing colorectal cancer screening intervention. Workshops may be followed up with in-depth interviews and observations if additional insights and perspectives from participants regarding the implementation of colorectal cancer screening are required. By actively involving stakeholders, the study aims to ensure that the collected information accurately reflects the realities of implementation and leads to actionable insights for improving screening services.

Participant selection

Participants will be selected using purposive sampling and snowballing techniques. The participants will be recruited from diff[illegible]re facilities and hospitals), geographic location[illegible]hern, east-coast, Sabah, and Sarawak[illegible]et population's participation ra[illegible]e, hospital appointment adherence, and[illegible]al cancer disease burden [7; provided in[illegible]60 participants, including various[illegible]involved in implementing the screeni[illegible]gram (e.g., medical officers, nurses, medical assistants, laboratory personnel, family medicine specialists, colorect[illegible]nterologists).

> Consumers of screening programs are vital stakeholders in the process. I apologise if I have missed your inclusion of consumers, however if you have not included them you should.

Group-based activities in workshops

At least two workshop sessions will be conduct[illegible] direct interaction and engagement among the participants. These sessions will gather heterogeneous groups of participants to obtain potential v[illegible]rocess. Additional sessions may be conducted in-person, virtu[illegible]g on the feasibility and participants' availability, especia[illegible]s is required.

> How many participants will be included in each workshop session?

> How do you plan to collect the data?

The participants will discuss and review the in[illegible]ing each workshop, specifically the first part of the session. The research team will develop this map following literature review and feedback from key stakeholders. Moderators will guide the sessions using an interview guide developed to gather the participants' perspectives regarding the implementation of colorectal cancer screening. The interview guide will be refined based on participant feedback and a thorough review of the information gathered during the study (provided in Annex 5 in S1 File). The valuable insights, specific process details, and discussions on areas for improvement provided by the participants will be carefully considered and incorporated into the process flow diagrams.

For the second part of the workshop, participants will engage in the concept of system support mapping and receive guidance in creating their system support maps. Participants will be asked to create a support map reflecting their specific colorectal cancer intervention activities. The map should include elements such as (a) their roles within the program, (b) critical responsibilities within each role, (c) needs for effective performance of each responsibility, (d) resources previously utilized for support, and (e) suggestions for individual or systematic support. Additionally, participants will consider (f) contextual factors that may influence their responsibilities and ability to fulfill them, considering differences in populations serviced and geographic regions. Moderators will guide and explain throughout the session to ensure clarity and understanding.

Following the individual mapping exercise, debriefing sessions will be conducted to encourage collective sense-making and reflection on implementation [illegible] Participants will have the opportunity to discuss their map[illegible] feedback, fostering a deeper understanding of the s[illegible] Participants may use papers and sticky notes to [illegible] mapping activity. The research team will then tra[illegible] such as Google Jamboard or other whiteboarding [illegible] Google Sheets, for further analysis and synthesis.

How will it be analysed? Using deductive coding according to a codebook designed on the basis of elements and categories identified in the literature review? Is there an intention to use inductive thematic analysis? Please provide more detail with quality references.

Data analysis

Data collected from the workshops will be analyzed to identify different steps and processes involved in implementing the screening intervention. The analysis will also seek to identify current processes, the variations and gaps, and proposed improvements in the implementation. The process flow diagrams will illustrate the sequential steps in implementing the colorectal cancer screening program and identify key stakeholders associated with each step, such as nurses, medical assistants, medical officers, specialists, laboratory personnel, endoscopy providers, and others.

Spelling

To analyze the system support [illegible] the breadth of entries for each element of the map. We will ten use the map entries for each element to identify themes, which will then be compared across the maps. After identifying themes for each element, we will consider the pathways (i.e., connections) between elements, from individual to systems level, with a particular focus on how and why participants selected their proposed improvements. Finally, we will use illustrative quotations to highlight and explain key themes.

Data integration

Data integration will occur at two points: During design and interpretation. Initially, quantitative data derived from routine monitoring and evaluation of the screening program will guide the participant selection for the qualitative component. Once quantitative and qualitative data have been independently gathered and analyzed, we will integrate the findings during interpretation. The trends and patterns observed in colorectal cancer screening uptake over time (quantitative data) will complement and contextualize information gathered from participant discussions (qualitative data).

Given the complexity and multidimensionality of data integration, we recognize that our interpretations may introduce bias. We plan to engage external stakeholders and experts actively to address this issue and ensure a comprehensive and unbiased synthesis of the results. Their valuable input and diverse perspectives will provide an important counterweight to our interpretations, contributing to a more robust analysis.

Data management and oversight

Access to the data will be limited to the research team members directly involved in data analysis and processing. Strict confidentiality m[illegible]wed to protect the participants' identities and sensitive informa[illegible]zed, and unique identifiers or pseudonyms will be assigned [illegible]dentiality during analysis and reporting. The final refined vers[illegible]will be stored in a designated repository in the Ministry of Health.

For how long?

Ethical approval and study registration

The study will comply with ethical principles outlined in the Dec[illegible]and Malaysian Good Clinical Practice Guideline. Ethical appro[illegible]dical Research and Ethics Committee, Ministry of Health (23-0[illegible]ered at the National Medical Research Register (NMRR ID-23-0[illegible]ata used in this study are collected in anonymized format. Data c[illegible]ection will span the study period, expected to be from September 1, 2023 to June 30, 2024 (Objectives 1 and 2). Workshops are scheduled between May 1, 2024 and November 30, 2024. Participation in the study is voluntary and participants will provide written informed consent (Objective 3).

So it is occurring now?

Knowledge translation through the Knowledge to Action framework

The study findings will be disseminated through established channels of knowledge sharing, such as stakeholder dialogue sessions, peer-reviewed journal articles, and presentations at scientific conferences. Knowledge translation is pivotal in bridging the gap between research and real-world application, ensuring that scientific insights lead to tangible health improvements. In guiding this transition, we apply the Knowledge to Action framework [33], emphasizing the dynamic and iterative relationship between knowledge creation and its practical application (see Fig 1).

Identifying the know-do gap. In the initial phase of the knowledge translation cycle, we aim to bridge the gap between existing research evidence and practical applications in the field. Complemented by the qualitative study, our comprehensive literature review lays the foundation for this. By comparing international best practices with current strategies and mapped processes, we will identify discrepancies and areas where improvements are needed in colorectal cancer screening.

Knowledge adaptation to local context. Our quantitative and qualitative analysis provides a detailed view of the current landscape of colorectal cancer screening, providing insights into regional disparities, access challenges, and variations in program

implementation. This empirical data serves as a guidepost, allowing us to tailor global best practices to fit the unique nuances and needs of our local context.

Assessment of barriers and facilitators to knowledge use. Our qualitative exploration gathers the perspectives of various stakeholders, identifying systemic, organizational, and individual factors that may either catalyze or impede the implementation of proposed strategies. These findings will be integral in designing context-sensitive interventions and attuning them to on-ground dynamics.

Intervention design and implementation. With a comprehensive understanding from our literature review, quantitative analysis, and qualitative insights, we will collaboratively develop and tailor interventions to improve colorectal cancer screening with key stakeholders. These strategies will address the identified know-do gaps while considering local conditions and potential barriers.

Monitoring and refinement. While a detailed monitoring plan is beyond the scope of this protocol, there will be a concerted effort to monitor how the introduced strategies are being adopted and implemented. Periodic assessment will be conducted to evaluate the uptake of recommendations and the tangible impact on colorectal cancer screening rates. Findings from the study may contribute to areas that need to be monitored and evaluated by program managers in the short term. Future work will involve working closely with stakeholders to refine and revise policies based on feedback and observed outcomes.

This cyclical process will ensure that the improvements in colorectal cancer screening are not transient but become an enduring part of the system. We aim to drive meaningful change that optimizes colorectal cancer screening processes and outcomes by embedding the knowledge translation framework into our research approach.

Discussion

This article outlines the design and methodological approaches of a multicomponent study aiming to address the issues of suboptimal screening participation in Malaysia's colorectal cancer screening program.

This study's short- and medium-term benefit lies in its potential to streamline the processes and optimize colorectal cancer screening in Malaysia, significantly improving public health outcomes based on a precision public health paradigm. This study will develop strategies that align the program with international best practices suitable to Malaysia's context [9]. It is incumbent upon the study to examine the complex interplay of factors at individual, community, and systemic levels that influence screening rates and explore pragmatic and innovative approaches to overcome these challenges. The identified barriers and challenges within the screening program, including logistical and coordination issues, program complexity, and socioeconomic inequalities, interact with one another. Addressing one aspect may impact others, highlighting the need for a comprehensive and integrated approach to optimize colorectal cancer screening, reduce health inequalities, and reduce the burden of colorectal cancer in the population [25].

Meanwhile, the long-term benefit of this study lies in its potential to position screening as a crucial entry point to the cancer care continuum, extending beyond colorectal cancer. By mapping available resources and understanding the demand for cancer treatment, we can utilize screening as a proxy for access to care. This approach allows us to identify gaps in the healthcare systems and strategically allocate scarce healthcare resources for colorectal cancer screening based on the population's healthcare needs to ensure early detection and timely and appropriate cancer treatment for those in need.

The significance of this study extends beyond the boundaries of the Malaysian healthcare system. The evidence-based insights and recommendations derived from this study have the potential to offer valuable lessons and pragmatic strategies that can be applied to international settings, particularly in low- and middle-income countries facing similar challenges. Disseminating the study's findings can inform future research endeavors

and policy development initiatives, contributing to global advancements in colorectal cancer screening. The significance of this study lies in its contributions to epidemiology through spatiotemporal analysis, its focus on equality and resource allocation, its bottom-up approach through stakeholder engagement, its review of process flow to address challenges, and its emphasis on translating evidence into actions.

The study is not without challenges. First, granularity constraints in spatiotemporal analysis. While spatiotemporal analysis can pinpoint geographic trends and disparities in areas with sparse data or limited reporting, the findings might not capture the complete picture. Additionally, this method captures associations but does not necessarily indicate causation, limiting the depth of insights derived from the quantitative data. This constraint can be addressed by including the qualitative component to understand the barriers, facilitators, and nuances of colorectal cancer screening. Second, while qualitative data provides detailed perspectives and contextual understanding, its inherently subjective nature may limit its applicability to broader populations. However, the insights we will gather and improvements suggested for implementing colorectal cancer screening can be adapted to other settings, provided specific contextual conditions are considered. Third, this study focuses on a colorectal cancer screening program implemented in the public health sector. We acknowledge the possibility of concurrent screening initiatives undertaken by the private health sector and non-governmental organizations. We will include these stakeholders in different study phases to mitigate potential oversights, ensuring a b[illegible] encompassing perspective on Malaysia's colorectal cancer sc[illegible]

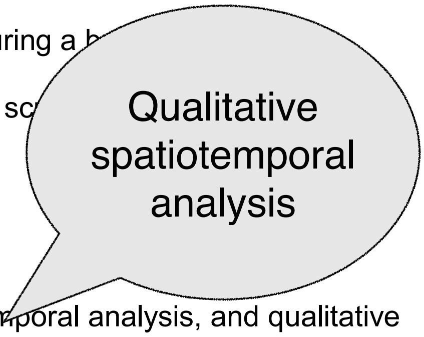

Conclusion

In summary, this study combines literature review, spatiotemporal analysis, and qualitative inquiry to gain a comprehensive understanding of colorectal cancer screening in Malaysia. By contrasting international best practices with local strategies, the study highlights discrepancies and provides data-driven recommendations for strategies tailored to the local context. By applying the knowledge-to-action framework, the study emphasizes continual

improvement and aims to optimize colorectal cancer screening implementation and contribute to improving population health outcomes.

Supporting information

S1 File. All tables are provided in Annexes 1-5.

Acknowledgments

The authors would like to thank the Director-General of Health Malaysia for permission to publish this paper.

Authors contributions

Conceptualization: Diane Woei-Quan Chong, Vivek Jason Jayaraj, Fathullah Iqbal Ab Rahim, Sharifah Saffinas Syed Soffian, Muhammad Fikri Azmi, Sondi Sararaks

Funding acquisition: Diane Woei-Quan Chong, Vivek Jason Jayaraj, Fathullah Iqbal Ab Rahim, Sharifah Saffinas Syed Soffian, Muhammad Fikri Azmi, Sondi Sararaks

Methodology: Diane Woei-Quan Chong, Vivek Jason Jayaraj, Fathullah Iqbal Ab Rahim, Sharifah Saffinas Syed Soffian, Muhammad Fikri Azmi, Sondi Sararaks

Data curation, analysis, and interpretation of data: Diane Woei-Quan Chong, Vivek Jason Jayaraj, Fathullah Iqbal Ab Rahim, Sharifah Saffinas Syed Soffian, Muhammad Fikri Azmi, Mohd Yusaini Mohd Yusri, Ahmad Shanwani Mohamed Sidek, Norfarizan Azmi, Rosaida Md Said, Muhammad Firdaus Md Salleh, Norasiah Abu Bakar, Hamiza Shahar, Rima Marhayu Abdul Rashid, Shazimah Abdul Samad, Zanita Ahmad, Mohd Safiee Ismail, Adilah A. Bakar, Nor Mashitah Hj Jobli, Sondi Sararaks

Project administration: Diane Woei-Quan Chong, Vivek Jason Jayaraj, Fathullah Iqbal Ab Rahim, Sharifah Saffinas Syed Soffian, Muhammad Fikri Azmi

Supervision: Sondi Sararaks

Writing – original draft: Diane Woei-Quan Chong

Writing – review & editing: Diane Woei-Quan Chong, Vivek Jason Jayaraj, Fathullah Iqbal Ab Rahim, Sharifah Saffinas Syed Soffian, Muhammad Fikri Azmi, Mohd Yusaini Mohd Yusri, Ahmad Shanwani Mohamed Sidek, Norfarizan Azmi, Rosaida Md Said, Muhammad Firdaus Md Salleh, Norasiah Abu Bakar, Hamiza Shahar, Rima Marhayu Abdul Rashid, Shazimah Abdul Samad, Zanita Ahmad, Mohd Safiee Ismail, Adilah A. Bakar, Nor Mashitah Hj Jobli, Sondi Sararaks

Competing interests

The authors have no conflict of interest to declare.

Financial disclosure statement

Funding for this research has been applied for through the Ministry of Health Malaysia Research Grant (MRG), with approval pending. The prospective funding body will have no involvement in the study design, data collection and analysis, publication decision, or manuscript preparation.

Data availability statement

Data used in this study are sourced from the Ministry of Health Malaysia and are not publicly available due to confidentiality restrictions. However, data can be accessed upon reasonable request and subject to approval from the relevant authority within the Ministry of Health Malaysia.

References

1. Xi Y, Xu P. Global colorectal cancer burden in 2020 and projections to 2040. Transl Oncol. 2021;14: 101174. doi:10.1016/j.tranon.2021.101174

2. U.S. Preventive Services Task Force. Screening for Colorectal Cancer: U.S. Preventive Services Task Force Recommendation Statement. Ann Intern Med. 2008;149: 627. doi:10.7326/0003-4819-149-9-200811040-00243

3. World Health Organization. Tackling NCDs: “best buys” and other recommended interventions for the prevention and control of noncommunicable diseases. World Health Organization; 2017. Report No.: WHO/NMH/NVI/17.9. Available: https://apps.who.int/iris/handle/10665/259232

4. Breekveldt ECH, Lansdorp-Vogelaar I, Toes-Zoutendijk E, Spaander MCW, Vuuren AJ van, Kemenade FJ van, et al. Colorectal cancer incidence, mortality, tumour characteristics, and treatment before and after introduction of the faecal immunochemical testing-based screening programme in the Netherlands: a population-based study. The Lancet Gastroenterology & Hepatology. 2022;7: 60–68. doi:10.1016/S2468-1253(21)00368-X

5. Brenner H, Chen C. The colorectal cancer epidemic: challenges and opportunities for primary, secondary and tertiary prevention. Br J Cancer. 2018;119: 785–792. doi:10.1038/s41416-018-0264-x

6. Zhang L, Mosquera I, Lucas E, Rol ML, Carvalho AL, Basu P, et al. CanScreen5, a global repository for breast, cervical and colorectal cancer screening programs. Nat Med. 2023;29: 1135–1145. doi:10.1038/s41591-023-02315-6

7. Ministry of Health. Malaysia National Cancer Registry Report (MNCR) 2012-2016. 2019. Available: https://www.moh.gov.my/moh/resources/Penerbitan/Laporan/Umum/2012-2016%20(MNCRR)/MNCR_2012-2016_FINAL_(PUBLISHED_2019).pdf

8. Ministry of Health. National strategic plan for colorectal cancer (NSPCRC) 2021-2025. 2021. Available:

https://www.moh.gov.my/moh/resources/Penerbitan/Rujukan/NCD/Kanser/National_Strategic_Plan_for_Colorectal_Cancer_(NSPCRC)_2021-2025.pdf

9. Tamin NSI, Razalli KA, Sallahuddin SN, Chan H-K, Hassan MRA. A 5-year evaluation of using stool-based test for opportunistic colorectal cancer screening in primary health institutions across Malaysia. Cancer Epidemiol. 2020;69: 101829. doi:10.1016/j.canep.2020.101829

10. Moss S, Ancelle-Park R, Brenner H. European guidelines for quality assurance in colorectal cancer screening and diagnosis. First Edition – Evaluation and interpretation of screening outcomes. Endoscopy. 2012;44: SE49–SE64. doi:10.1055/s-0032-1309788

11. Robertson DJ, Lee JK, Boland CR, Dominitz JA, Giardiello FM, Johnson DA, et al. Recommendations on fecal immunochemical testing to screen for colorectal neoplasia: A consensus statement by the US multi-society task force on colorectal cancer. Am J Gastroenterol. 2017;112: 37–53. doi:10.1038/ajg.2016.492

12. Bujang N-N-A, Lee Y-J, Mohd-Zain S-A-S, Aris J-H, Md-Yusoff F-A, Suli Z, et al. Factors associated with colorectal cancer screening via immunochemical fecal occult blood test in an average-risk population from a multiethnic, middle-income setting. JCO Global Oncology. 2021; 333–341. doi:10.1200/GO.20.00460

13. Chan MW, Chean KY, Kader Maideen SF, Kow FP. The intention and uptake of colorectal cancer screening after a brief health education program in a malaysian primary care setting: A population-based study. Asian Pac J Cancer Prev. 2021;22: 3475–3482. doi:10.31557/APJCP.2021.22.11.3475

14. Doraimuthu S, Dahlui M, Hoe VCW, Su TT. Exploration of Malaysian stakeholders view on barriers to and facilitators of colorectal cancer screening among older population. Asia Pac J Public Health. 2023;35: 27–33. doi:10.1177/10105395221145786

15. Coronado GD, Retecki S, Petrik AF, Coury J, Aguirre J, Taplin SH, et al. Mapping multi-site clinic workflows to design systems-enabled interventions. 2017;5.

16. Schliemann D, Ramanathan K, Matovu N, O'Neill C, Kee F, Su TT, et al. The implementation of colorectal cancer screening interventions in low-and middle-income countries: a scoping review. BMC Cancer. 2021;21: 1125. doi:10.1186/s12885-021-08809-1

17. Priaulx J, Turnbull E, Heijnsdijk E, Csanádi M, Senore C, de Koning HJ, et al. The influence of health systems on breast, cervical and colorectal cancer screening: an overview of systematic reviews using health systems and implementation research frameworks. J Health Serv Res Policy. 2020;25: 49–58. doi:10.1177/1355819619842314

18. Turnbull E, Priaulx J, de Kok IMCM, Lansdorp-Vogelaar I, Anttila A, Sarkeala T, et al. Results of a health systems approach to identify barriers to population-based cervical and colorectal cancer screening programmes in six European countries. Health Policy. 2018;122: 1206–1211. doi:10.1016/j.healthpol.2018.08.005

19. Fedewa SA, Cullati S, Bouchardy C, Welle I, Burton-Jeangros C, Manor O, et al. Colorectal cancer screening in Switzerland: cross-sectional trends (2007-2012) in socioeconomic disparities. PLoS One. 2015;10: e0131205. doi:10.1371/journal.pone.0131205

20. Fidler MM, Bray F. Global cancer inequalities. Front Oncol. 2018;8: 293. doi:10.3389/fonc.2018.00293

21. Frederiksen BL, Jørgensen T, Brasso K, Holten I, Osler M. Socioeconomic position and participation in colorectal cancer screening. Br J Cancer. 2010;103: 1496–1501. doi:10.1038/sj.bjc.6605962

22. Tatari CR, Andersen B, Brogaard T, Badre-Esfahani SK, Jaafar N, Kirkegaard P. Perceptions about cancer and barriers towards cancer screening among ethnic minority women in a deprived area in Denmark – a qualitative study. BMC Public Health. 2020;20: 921. doi:10.1186/s12889-020-09037-1

23. Carethers JM, Doubeni CA. Causes of socioeconomic disparities in colorectal cancer and intervention framework and strategies. Gastroenterology. 2020;158: 354–367. doi:10.1053/j.gastro.2019.10.029

24. Song EY. Colorectal cancer risk factors and screening among the uninsured of tampa bay: a free clinic study. Prev Chronic Dis. 2021;18. doi:10.5888/pcd18.200496

25. O'Leary MC. Pragmatic evidence-based decision-making: Using systems science tools to inform implementation of colorectal cancer screening interventions. University of North Carolina. 2022.

26. Adam T, de Savigny D. Systems thinking for strengthening health systems in LMICs: need for a paradigm shift. Health Policy and Planning. 2012;27: iv1–iv3. doi:10.1093/heapol/czs084

27. Carey G, Malbon E, Carey N, Joyce A, Crammond B, Carey A. Systems science and systems thinking for public health: a systematic review of the field. BMJ Open. 2015;5: e009002. doi:10.1136/bmjopen-2015-009002

28. O'Leary MC, Hassmiller Lich K, Frerichs L, Leeman J, Reuland DS, Wheeler SB. Extending analytic methods for economic evaluation in implementation science. Implementation Sci. 2022;17: 27. doi:10.1186/s13012-022-01192-w

29. Wheeler SB, Leeman J, Hassmiller Lich K, Tangka FKL, Davis MM, Richardson LC. Data-powered participatory decision making: Leveraging systems thinking and simulation to guide selection and implementation of evidence-based colorectal cancer

screening interventions. Cancer J. 2018;24: 136–143. doi:10.1097/PPO.0000000000000317

30. O'Cathain A, Murphy E, Nicholl J. The quality of mixed methods studies in health services research. Journal of health services research & policy. 2008;13: 92–8. doi:10.1258/jhsrp.2007.007074

31. Clegg LX, Hankey BF, Tiwari R, Feuer EJ, Edwards BK. Estimating average annual per cent change in trend analysis. Statistics in Medicine. 2009;28: 3670–3682. doi:10.1002/sim.3733

32. Hyndman RJ, Athanasopoulos G. Forecasting: Principles and Practice. 2nd Editio. Monash University Australia; 2018.

33. Graham ID, Logan J, Harrison MB, Straus SE, Tetroe J, Caswell W, et al. Lost in knowledge translation: Time for a map? Journal of Continuing Education in the Health Professions. 2006;26: 13–24. doi:10.1002/chp.47

Figure

Click here to access/download;Figure;Fig1.tiff

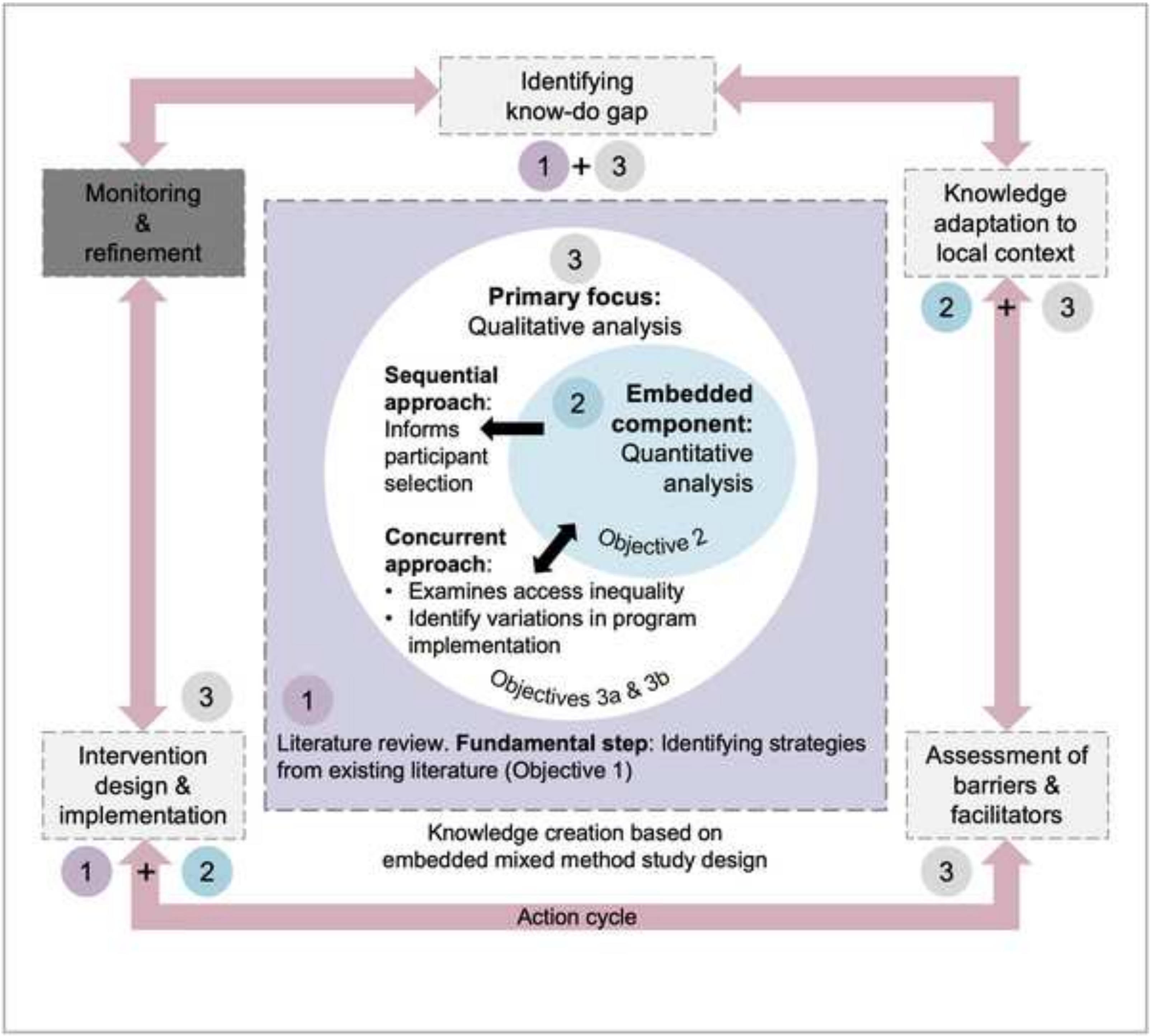

Click here to access/download
Supporting Information
S1 File.docx
